# Special Issue “Molecular Biology of the Thyroid Cancer and Thyroid Dysfunctions”

**DOI:** 10.3390/ijms262311586

**Published:** 2025-11-29

**Authors:** Lavinia Vija

**Affiliations:** 1 Department of Nuclear Medecine, Oncopole Claudius Regaud, 1 Avenue Irène Joliot Curie, CEDEX 9, 31059 Toulouse, France; vija.lavinia@iuct-oncopole.fr; 2 Biophysics and Nuclear Medicine, Toulouse University Medical School, 133, Route de Narbonne, CEDEX 9, 31062 Toulouse, France

Thyroid disorders (comprising thyroid dysfunctions such as hypo- or hyperthyroidism and thyroid cancer) are among the most common endocrine pathologies. Thyroid cancer presents increasing incidence rates but low mortality which is explained by the detection of papillary thyroid cancers at a younger age, mainly as early-stage papillary thyroid cancers [1].

Due to the increasing number of diagnosed thyroid cancers, new challenges have arisen. Determining methods for the better identification of biomarkers to screen thyroid nodules in order to reduce the number of thyroid resections has become a public health issue. New types of molecular biomarkers based on multigene genomic classifiers on fine-needle aspiration samples are currently being explored all over the world in order that we might better distinguish benign from malignant thyroid nodules or better guide surgery for cytologically indeterminate thyroid nodules [2,3,4]. However, this complex approach has revealed a certain heterogeneity and a notable lack of precision; the negative predictive value is not high enough to avoid surgery in indeterminate thyroid nodules.

The management of adult patients with differentiated thyroid cancer was recently updated with new recommendations published in 2025 by the American Thyroid Association [5]. A new risk stratification classification addressing not only papillary but also follicular and oncocytic thyroid carcinoma was proposed with several re-escalade tendencies, redefining the first line therapies in differentiated thyroid cancer. First line therapy with targeted drugs against RET, NTRK and ALK fusions was also strongly recommended in patients with iodine refractory thyroid cancer harboring this particular molecular profile, highlighting the need of molecular biology tests and detection of mutations in iodine refractory metastatic thyroid tissue. 

This first edition of “Molecular Biology of the Thyroid Cancer and Thyroid Dysfunctions” aimed at expanding our knowledge of the molecular mechanisms of thyroid disorders and explored particular elements of the thyroid microenvironment, gathering nine original manuscripts and three reviews.

I wish to express my sincere gratitude to all authors, to peer reviewers, and to the complete editorial team whose contributions, quick replies and availability have made this Special Issue possible. Together, we managed to improve the scientific inquiries and lead a collaborative effort to identify interesting complex particular aspects related to thyroid dysfunctions.

This Special Issue organized the manuscripts into three major themes: pharmacological potential in hyperthyroidism, molecular biomarkers of thyroid cancer aggressiveness and tumor microenvironment.

Thyroid hormone excess may cause complex systemic symptoms, determine cardiac complications such as atrial fibrillation and also lead to a rare but serious state related to thyroid storm, which requires both cardiac and thyroid control with beta blockers [6,7]. A review of beta-blockers’ utility, mechanisms of action and indications in thyroid pathologies, such as hyperthyroidism during prengnacy or hyperthyroidism induced by amiodarone, was published in this issue (contribution 1).

The availability of next-generation sequencing techniques used mainly to screen mutations in metastatic or iodine refractory thyroid cancer has significantly increased the identification of mutations—primarily in the MAP kinase pathway, BRAF, and RAS, but also in PIK3CA, TERT promoter, and TP53, with these last examples associated with rapid progression and worse outcomes. The determining events in the majority of papillary or follicular thyroid cancers are often related to clonal selection and mutation in single genes.

Thyroid oncogenesis is more complex. Recently, Hanahan defined as “hallmarks of cancer” many biological processes that could be involved in cancer progression and dedifferentiation, such as mismatch repair; gene instability; epigenetic reprogramming influencing cells populating the tumor microenvironment; or deregulations in cellular metabolism (i.e., mitochondrial and lipid metabolism) [8,9].

An update to the molecularly complex pathways determining thyroid oncogenesis was the subject of contribution 2. The interplay between long, non-coding RNAs and epigenetic modifications was also narratively reviewed in contributions 3 and 4.

Epigenetic features related to the TSHR methylation pattern and a selection of microRNAs interfering with TSH receptor gene methylation were explored in plasma samples from patients with papillary thyroid cancer and compared to healthy controls (contribution 5).

Other molecular biomarkers of aggressiveness were also explored in a study of miRNA and ETS1 protein expression (contribution 6).

Characterization of specific mutations allow a better understanding of particular complex syndromes associating struma ovarii with thyroid cancer (contribution 7) or familial oncocytic papillary thyroid carcinomas (contribution 8).

A panel of molecular biomarkers related to altered MAPK signaling or cell proliferation and their predictive or prognostic dimension was also explored in patients with thyroid cancers (contributions 9 and 10).

A series of molecular biomarkers with immunomodulatory roles in the tumoral microenvironment can be transferred from one cell to another via the extracellular vesicles (EVs). Several studies have explored EV-based biomarkers in thyroid diseases and thyroid cancers, presenting heterogeneous and often discordant results related to the different purification techniques and analytical and pre analytical procedures [10].

Employing tissue on chip analysis, EVs derived from the thyroid cells of patients with benign thyroid, Graves’ disease, and thyroid cancer were studied. This analysis can provide information on the bioactive cargo of several miRNAs and compare the particularities of the EVs with respect to different thyroid disorders (contribution 11).

Obesity is another public health problem linked to cardiovascular risk and cancer. Several research teams have explored the links between chronic inflammation, adipokine expression, hyperinsulinemia, estrogens and altered immune response factors explaining the epidemiological increased risk of thyroid cancer in people with a high body mass index [11].

Dysregulations of adipokine production and the inflammation markers produced and regulated by the innate and adaptive immune cells organizing the tumor microenvironment could eventually link obesity or body mass index to papillary thyroid carcinoma. A small retrospective cohort of 238 patients with PTC was explored and classified according to their BMI. Authors did not manage to identify a link between the immune system and BMI, emphasizing the complexity and heterogeneity of the tumor microenvironment (contribution 12).

The articles collected in this Special Issue highlight the diversity of the current research landscape concerning the identification of biomarkers for thyroid cancer diagnosis and aggressiveness.

We hope that the insights into molecular biology and the complexity of thyroid cancer/dysfunctions presented in this Special Issue—the first, we hope, of multiple editions—will inspire further innovation and lead to new collaborations and fresh perspectives on the identification of interesting molecular biomarkers with potential roles to play in patient management.
